# The case for the introduction of new chemotherapy agents in the treatment of advanced non small cell lung cancer in the wake of the findings of The National Institute of Clinical Excellence (NICE)

**DOI:** 10.1038/sj.bjc.6600491

**Published:** 2002-08-27

**Authors:** J S Waters, M E R O'Brien

**Affiliations:** Lung Unit, The Royal Marsden Hospital, Sutton, Surrey, UK; Kent Cancer Centre, Maidstone, Kent, UK

**Keywords:** Lung cancer, chemotherapy, non small cell lung cancer, NICE

## Abstract

After years of nihilism towards the use of chemotherapy for non small cell lung cancer in the UK it would appear that we have now reached the point where the use of chemotherapy to relieve symptoms, maintain quality of life, and prolong life, are now accepted for informed patients with good performance status willing to accept short-term toxicities. The use of the new agents vinorelbine, gemcitabine and paclitaxel in combination with cisplatin or carboplatin are all active regimens which offer small but real advantages over standard UK triple therapies (MVP, MIC) in terms of resource use, toxicity profiles and response rates. Overall survival could be increased by as much as 10% at one year on indirect comparisons. The use of docetaxel as second line therapy now offers lung cancer patients a second bite of the cherry, and should overall also prolong survival. It is only in embracing these small gains that we can currently make progress in the treatment of NSCLC.

*British Journal of Cancer* (2002) **87**, 481–490. doi:10.1038/sj.bjc.6600491 www.bjcancer.com

© 2002 Cancer Research UK

## 

The widespread nihilism in the UK towards palliative lung cancer treatment has meant that patients with all stages of lung cancer have been deprived of treatment that adds small survival gains and improved symptom relief. This may contribute to the overall dismal outcome survival figures for lung cancer in the UK (Janssen-Heijnen *et al*, 1998). At this time chemotherapy is sufficiently active to justify its use in patients of good performance status who understand the true goals of chemotherapy and its potential toxicities – but what should we use? The current questions regarding palliative chemotherapy in the UK remain – is any doublet of the new or old generation better than UK triple therapy for patients of performance status zero or one, and can single agent chemotherapy replace UK triple therapy for patients of performance status two? This review will examine the data in support of the new agents that have been assessed by the National Institute of Clinical Excellence in an attempt to answer these questions.

## CURRENT STANDARDS

The commonest (standard) treatments for non-small cell lung cancer (NSCLC) in this country are cisplatin based, usually MVP (mitomycin C, vinblastine and cisplatin) or MIC (mitomycin C, iphosphamide and cisplatin) with cisplatin used at a dose of around 50–60 mg m^−2^. This differs to the United States where the cisplatin dose is usually higher and the combinations most frequently used until the late 1990s were etoposide plus cisplatin and vinblastine or vindesine with cisplatin. These regimens have been directly compared in several trials. One of these trials compared MIC, MVP and etoposide/cisplatin and showed a significant survival advantage for both three drug regimens (median survival 36 weeks, 42 weeks, and 27 weeks respectively; *P*<0.04) (Crino *et al*, 1995). Other randomised trials did not report any advantages with the cisplatin triplets (Ruckdeschel *et al*, 1986; Crino *et al*, 1990).

The use of higher cisplatin doses was based on one small randomised trial involving 85 patients, that demonstrated a longer duration of response and a survival advantage for responders in the higher dose arm but overall survival was not reported (Gralla *et al*, 1981). However, three subsequent larger randomised studies have failed to show an advantage for the higher cisplatin doses and toxicity was considerably worse (Table 1

**Table 1:**
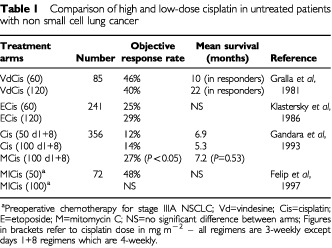
Comparison of high and low-dose cisplatin in untreated patients with non small cell lung cancer

) (Gandara *et al*, 1993; Klastersky *et al*, 1986; Felip *et al*, 1997). Thus UK oncologists tend to use cisplatin at a dose of 50 mg m^−2^ in triple therapy given three-weekly, and this is also well tolerated by performance status-two patients (Smith *et al*, 2001).

The standard duration of chemotherapy has also recently been challenged. A trial of MVP chemotherapy showed that three courses produced the same symptomatic benefits and response rates as six. Haematological toxicity increased during courses four to six in patients randomised to receive six courses with grade 3–4 leucopenia in 22% and grade 3–4 thrombocytopenia in 7% compared with 12% and 1% respectively in the patients randomised to receive three courses (Smith *et al*, 2001). The median survival was 6 *vs* 7 months in the two arms, and the 1-year survival was 22% *vs* 25% (*P*=0.2). In the subgroup of patients of performance status 0 and 1 the 1-year survival was no different at 25.6% (95% CI, 20.8–32.5%), and performance status did not come out as an independent prognostic factor in this trial. Quality of life deteriorated after the fourth course in the group having six courses of chemotherapy. These results were corroborated by a recently reported trial comparing treatment with a combination of carboplatin and paclitaxel for four cycles or continued until disease progression. Survival and quality of life were the same in the two treatment arms, as was the median number of chemotherapy cycles delivered (Socinski *et al*, 2001). Most centres now treat NSCLC with 3–4 courses of palliative chemotherapy. This of course has major health economic implications.

In patients who are not suitable for cisplatin based treatment, carboplatin can be used instead on the basis that for most solid tumours carboplatin produces similar palliative benefits to cisplatin. The three-drug combination mitomycin C, vinblastine and carboplatin (MVCarbo) is given as a day case, and appears to be as active as MVP but with more myelosuppression, with rates of grade 3–4 leucopenia of 24% and grade 3–4 thrombocytopenia of 22% (Gregory *et al*, 2001). The drug costs for MVCarbo are about the same as for single agent vinorelbine or gemcitabine. Two randomised trials have addressed whether carboplatin can replace cisplatin in standard lung cancer chemotherapy regimens. The first trial, conducted by the EORTC, evaluated cisplatin or carboplatin in combination with etoposide, using a cisplatin dose of 120 mg m^−2^ and a carboplatin dose of 325 mg m^−2^ (Klastersky *et al*, 1990). Two hundred and twenty-eight eligible patients were randomised among whom response rates were 16% in the carboplatin arm and 27% in the patients treated with cisplatin (*P*=0.07). No survival difference was demonstrated (median survival 27 *vs* 30 weeks; *P*=0.35), and higher rates of toxicity were seen with the cisplatin regimen. In the second trial involving 221 patients, the regimen investigated was mitomycin, vinblastine and platinum, using either cisplatin 120 mg m^−2^ or carboplatin 500 mg m^−2^ (Jelic *et al*, 2001). Thus the carboplatin : cisplatin dose ratio was greater in this study. Response rates were identical in the two arms (37% *vs* 36%; *P*>0.9), but both progression-free survival (*P*=0.005) and overall survival (*P*=0.008) favoured the carboplatin regimen. The authors do not report median survival times or the proportion of patients surviving for 1 year and it is therefore difficult to ascertain the clinical relevance of this difference. Furthermore, a significant imbalance between the two arms was found in relation to performance status, with a significantly greater proportion of performance status 0 and 1 patients in the carboplatin arm. In this trial the carboplatin regimen produced significantly more haematological toxicity whereas the cisplatin regimen was more emetogenic. On a cautionary note, a numerically inferior survival was produced by a regimen of carboplatin and docetaxel compared with a cisplatin/docetaxel combination (median survival 9.1 months *vs* 10.9 months). However, this randomised trial was designed to compare each of these regimens with a control arm of cisplatin plus vinorelbine and was not powered to examine the difference between the two docetaxel-containing arms (Rodriguez *et al*, 2001). The carboplatin/cisplatin debate goes on and a trial to definitively answer this question will need careful design and large numbers of patients.

## BACKGROUND TO THE NEW DRUGS

Gemcitabine, the taxanes (paclitaxel, docetaxel), and vinorelbine all have significant single agent activity with response rates of at least 20% and encouraging survival data with acceptable toxicities. It is gratifying to see that they have all been tested against best supportive care in NSCLC (Table 2

**Table 2:**
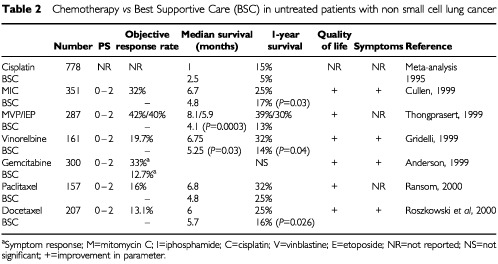
Chemotherapy *vs* Best Supportive Care (BSC) in untreated patients with non small cell lung cancer

). Similarly all four drugs have been combined with cisplatin with predictable toxicity and higher response rates than are achieved with cisplatin alone – this data will not be enlarged upon as single agent cisplatin is not a usual therapy in the UK. On the other hand there is little data on the use of a new agent with cisplatin versus a new agent alone, vinorelbine being the drug with the most data on this issue to date. However a recently completed study in the US compared paclitaxel plus cisplatin with single agent paclitaxel, and the results are awaited with interest (Lilenbaum *et al*, 2001). More recently a number of doublets have been compared with each other in randomised trials.

## CLINICAL EFFECTIVENESS OF VINORELBINE

### Vinorelbine *vs* best supportive care

A large randomised phase III trial, compared vinorelbine to best supportive care alone in 191 elderly patients (over 70 years) with NSCLC stages IIIb or IV, WHO performance status 0–2 (Elderly Lung Cancer Vinorelbine Italian Study Group, 1999). The primary end point was improvement in quality of life. Secondary endpoints were toxicity and tumour response in the vinorelbine arm. Vinorelbine treated patients had an improved quality of life, measured as improvement in physical, cognitive and social functioning compared to patients treated with best supportive care only. Treated patients also recorded improvements in disease related symptoms (cough, dyspnoea, pain, fatigue) but worse toxicity-related symptoms (constipation, nausea and vomiting, peripheral neuropathy and hair loss). Survival rates for the vinorelbine-treated patients at 6 and 12 months were 55% and 32%, compared to 41% and 14% in the control group. Median survival increased from 21 to 28 weeks (*P*=0.03). After adjustment for stage of disease and performance status, the estimated relative hazard of death for the vinorelbine-treated patients was 0.65 (95% confidence interval [CI], 0.45–0.93) compared to those in the control arm. The objective overall response rate (ORR) in the vinorelbine-treated patients was 19.7% with a further 30.3% having stable disease, while 42.1% had progressive disease. Vinorelbine treatment was generally well tolerated by the majority of patients. Only five patients stopped treatment due to toxicity (Elderly Lung Cancer Vinorelbine Italian Study Group, 1999) (Table 2).

A similar study of 211 patients with stage IV NSCLC randomised patients to either vinorelbine or a control arm of 5-fluorouracil (5-FU) on a 2 : 1 ratio. The FDA chose the control arm of 5-FU, as it was not considered ethical to randomise against no treatment. The primary endpoints were survival and quality of life with response rates the secondary end point. The outcome favoured vinorelbine in terms of both median survival (30 *vs* 22 weeks, *P*<0.03) and the proportion of patients alive at 1 year (25% *vs* 16%) (Crawford *et al*, 1996).

### Vinorelbine in combination with cisplatin* vs* other standards

Vinorelbine plus cisplatin has been compared with single agent vinorelbine. In a three-arm study, 612 patients of performance status 0, 1, and 2 were randomised between vinorelbine/cisplatin, vindesine/cisplatin and vinorelbine alone. The vinorelbine/cisplatin combination resulted in a significantly superior response rate and survival (Table 3

**Table 3:**
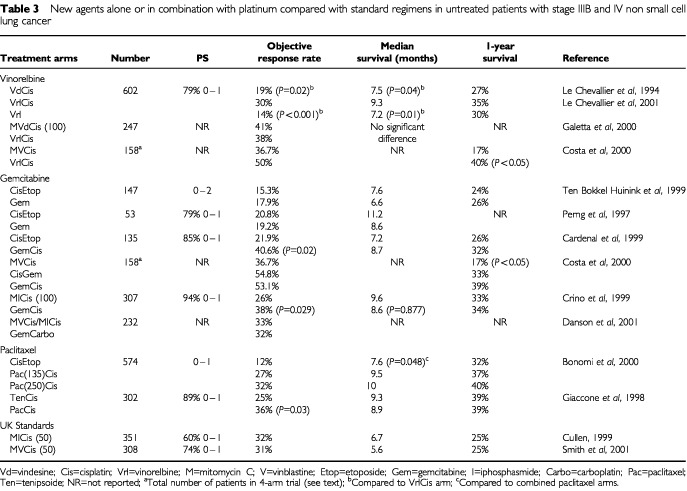
New agents alone or in combination with platinum compared with standard regimens in untreated patients with stage IIIB and IV non small cell lung cancer

) (Le Chevalier *et al*, 1994). More recent analysis of this data has shown that the subgroup of patients with performance status 2 had more toxicity and less benefit from vinorelbine and cisplatin when compared to vinorelbine alone (Le Chevalier *et al*, 2001). Two smaller randomised studies compared vinorelbine/cisplatin to vinorelbine alone and showed a non significant trend to improved survival: 41 *vs* 33 weeks (Gil Deza *et al*, 1996), and 33 *vs* 32 weeks (Depierre *et al*, 1999).

Two randomised clinical trials have compared vinorelbine plus cisplatin with standard cisplatin-based triplets. An Italian group has recently updated their study and presented it at the World Lung Cancer Meeting in Japan in September 2000. A total of 247 patients have been randomised to cisplatin and vinorelbine (VrlCis) or MVdP (cisplatin 100 mg m^−2^, vindesine and mitomycin C). There was no statistically significant difference between response rates (38% VrlCis and 41% MVdP), time to progression or overall survival. The VrlCis arm had more phlebitis and the MVdP arm had more myelosuppression and thrombocytopenia. The authors concluded VrlCis was as active as MVdP but less toxic (Table 3) (Galetta *et al*, 2000).

A Portuguese group have reported on the first 158 patients of a planned 240 entered in a randomised Phase III trial comparing MVP (mitomycin, vinblastine, cisplatin) *vs* VrlCis (vinorelbine and cisplatin) *vs* CisGem (cisplatin d1 and gemcitabine) *vs* GemCis (gemcitabine and cisplatin d15 and q28). The results are detailed in Table 3, demonstrating the superior activity of cisplatin combined with vinorelbine or gemcitabine *vs* MVP. However, this study was reported early and may well be underpowered to show a survival benefit. Further details of the patient population are also required (Costa *et al*, 2000).

## CLINICAL EFFECTIVENESS OF GEMCITABINE

Gemcitabine was launched in 1995 – the same year as the meta-analysis (Non-Small Cell Lung Cancer Collaborative Group, 1995). Phase II studies in NSCLC showed both activity and symptom control. An overall response rate of 20% was observed in the Manchester/Copenhagen study (and verified by an independent oncology review board), and 51 out of 73 patients (70%) reported an improvement in their tumour related symptoms (Anderson *et al*, 1994). Single agent gemcitabine has been studied in the elderly with advanced NSCLC (Pasquini *et al*, 1998; Ricci *et al*, 2000). Response rates and toxicity profiles were no different from other phase II studies of single agents in younger patients.

### Gemcitabine *vs* best supportive care

There is only one phase III study looking at single agent gemcitabine and comparing it to best supportive care (Anderson *et al*, 2000). This study randomised 300 patients and had as endpoints the degree and durability of symptom response. The response rate to gemcitabine was 19% (95% CI, 13–27%). Symptom response in the two arms was assessed at 2, 4, and 6 months and was significantly better in the gemcitabine treated patients at 2 and 4 months (*P*=0.048 and *P*=0.034) but was no different at 6 months. There was a trend to improved QoL in the gemcitabine arm with deterioration in the control arm over the first 2-month period, and a significant reduction in the need for radiotherapy – 49% for gemcitabine-treated patients *vs* 79% for controls. The most impressive result in this study was the difference in the number of days in hospital between the two study arms. Patients receiving best supportive care required on average 2012 days in hospital and 247 in hospice compared with 1746 days in hospital and 183 days in hospice for gemcitabine treated patients. There was no difference in survival between the treatment arms in this study but the study was underpowered to show this (Table 2).

### Gemcitabine *vs* other standards

A Taiwanese trial and a separate European study compared gemcitabine alone to etoposide/cisplatin. The response rates and survival were similar in both arms of both trials but the toxicity profile and inpatient days were markedly better in the gemcitabine arm (Perng *et al*, 1997; ten Bokkel Huinink *et al*, 1999). Similarily gemcitabine has been compared to cisplain and vindesine and again the single agent was less toxic and as effective (Vansteenkiste *et al*, 2000). These studies suggest gemcitabine can replace the older cisplatin doublets in the palliative treatment of NSCLC – we do not know if it can replace triplets including cisplatin and mitomycin C.

### Gemcitabine in combination with cisplatin* vs* other standard regimens

Gemcitabine/cisplatin is more active than cisplatin alone with a response rate of 30.4% *vs* 11.1% (*P*<0.0001) and a median survival of 9.1 months versus 7.6 months. However, the combination is more toxic for all haematological parameters (Sandler *et al*, 2000). A Spanish study has compared gemcitabine/cisplatin (GemCis) to cisplatin/etoposide (Cis/Etop). This study had both quality of life and pharmacoeconomic analyses but for a phase III study the number of patients included was small at 135 in total. The response rate was higher with the new combination (40.6% *vs* 21.9%, *P*=0.02), the median duration of response was prolonged by 6 weeks but the overall survival was not different and the authors concluded the trial was underpowered to show a survival difference (Table 3). Neutropenia was more common with Cis/Etop while thrombocytopenia was more common with GemCis. The quality of life showed no difference between the two treatments (Cardenal *et al*, 1999) and pharmacoeconomic data is also available (see cost effectiveness section) (Sacristan *et al*, 2000).

The most relevant trials for the UK are the comparisons of GemCis/GemCarbo to MIC/MVP. There are now three completed studies and a fourth in which this comparison is part of the four arm design. In the first reported by an Italian group (Table 3) (Crino *et al*, 1999), the MIC used differed from the UK MIC in that the cisplatin dose was 100 mg m^−2^ every 4 weeks while in the UK the dose is 50–60 mg m^−2^ every 3 weeks. There were 307 patients randomised in this study. The overall response rate was 38% for GemCis *vs* 26% for MIC (*P*=0.029). The median survival time was 8.6 months *vs* 9.6 months respectively (*P*=0.877). The incidence of severe neutropenia and anaemia was the same for both arms but there was more severe thrombocytopenia with the GemCis treatment, 64% *vs* 28% (*P*<0.001) and more alopecia with MIC, 12% *vs* 39% (*P*<0.001). The quality of life results were not different between the two treatment arms. Surprisingly this has lead to a change in practice in a number of European countries with widespread usage of gemcitabine and cisplatin.

A UK study reported in abstract form compared a carboplatin/gemcitabine regimen with MIC or MVP (Table 3) (Danson *et al*, 2001). At the time of this report, the outcome of 232 patients was analysed, although over 300 patients had been enrolled in the trial. Response rates were equivalent in the two arms (32% *vs* 33%) with rates of stable disease slightly higher in the triplet arm (26% *vs* 39%). Toxicity was reported to be similar between the two groups although the gemcitabine/carboplatin arm produced higher rates of haematological toxicity (*P*=0.006). Survival data are awaited. The third study addressing this question is also from the UK and has just been completed by the London Lung Group. Again data should be available this year. Finally, as discussed in the section on vinorelbine above, two different schedules of cisplatin and gemcitabine were demonstrated to produce higher response rates than MVP in a small four arm Portuguese trial (Table 3) (Costa *et al*, 2000).

## CLINICAL EFFECTIVENESS OF PACLITAXEL

### Paclitaxel *vs* best supportive care

In one UK based trial 157 patients with advanced NSCLC, newly diagnosed, with performance status 0, 1 or 2 were randomised to single agent paclitaxel or best supportive care. The primary endpoint was survival and this was statistically significantly improved in the patients receiving paclitaxel with a median survival of 6.8 months in the paclitaxel arm and 4.8 months in the best supportive care arm (*P*=0.037). Quality of life was similar for both treatment arms apart from the functional activity score of the Rotterdam Symptom Checklist which was statistically in favour of the paclitaxel arm (*P*=0.043) (Table 2) (Ranson *et al*, 2000).

### Paclitaxel in combination with cisplatin *vs* other standard regimens

The first of the studies using paclitaxel (Taxol) in combination with cisplatin started to appear around 1996. The ECOG study compared two doses of paclitaxel (250 and 135 mg m^−2^) with cisplatin to etoposide/cisplatin (Bonomi *et al*, 2000). Both paclitaxel/cisplatin doses resulted in similar response rates, which were higher than the etoposide/cisplatin arm (32% *vs* 27% *vs* 12%). The median survival was extended by about 2 months (10 *vs* 9.6 *vs* 7.7 months, *P*=0.048) in the paclitaxel/cisplatin arms with an improvement in the 1-year survival (39% *vs* 37% *vs* 32%). The EORTC conducted a two-arm study comparing cisplatin and paclitaxel to cisplatin and teniposide and although the overall response rate was higher with the paclitaxel treatment (36% vs 25%, *P*=0.03), there was no survival difference between the two arms. The quality of life was superior in the paclitaxel arm at 6 weeks but this was lost at 12 weeks (Giaccone *et al*, 1998).

## CLINICAL EFFECTIVENESS OF DOCETAXEL

Although NICE did not evaluate docetaxel as a first line treatment option, some data exists regarding its use in this setting. For completeness this is reviewed below.

### Docetaxel *vs* best supportive care

There is only one phase III study looking at single agent docetaxel and comparing it to best supportive care (Roszkowski *et al*, 2000). This study randomised 207 patients to either docetaxel at a dose of 100 mg m^−2^ or best supportive care. Patients of performance status 0, 1, and 2 were all included. The response rate to docetaxel was 13.1% (95% CI, 7.5–18.8%) and 19.6% in the evaluable patients. Symptom response (pain and dyspnoea) was significantly better in the docetaxel treated patients with less use of opiate analgesics (*P*<0.001) and less need for palliative radiotherapy (*P*<0.01). There was a trend to improved quality of life in the docetaxel arm for global health and physical functioning scores. The emotional functioning was significantly in favour of docetaxel (*P*=0.01). There was a statistically significant improvement in median survival in docetaxel-treated patients, with a median survival of 6 months compared to 5.7 months (*P*=0.026). This translated to an improved 1-year survival rate of 25% compared to 16% with best supportive care. At 2 years, 12% of the docetaxel patients were alive whereas none remained alive after 20 months in the best supportive care arm (Table 2).

### Docetaxel in combination with cisplatin *vs* other standard regimens

There is an ongoing trial comparing docetaxel plus cisplatin with the standard regimens MVP or MIC in the UK. This combination has been more extensively investigated in trials comparing the different new doublet regimens, and will be discussed below.

## TRIALS COMPARING THE NEW DRUGCOMBINATIONS

The favourable results with the newer agents discussed above has led to these ‘platinum/new agent doublets’ being adopted as standard chemotherapy regimens for advanced NSCLC in the United States and in parts of Europe. Several trials have now been conducted to examine whether any of these regimens stands out as being superior to the rest. The majority of these trials have only been the subject of reports in abstract form, and the mature results are awaited. However, in general, little difference in efficacy has been observed between the regimens studied, while differences in toxicity have largely been qualitative rather than quantitative.

### Comparisons of platinum-containing doublets

At ASCO 2000 the first results of the long awaited ECOG 1594 trial were reported. This is one of the biggest trials in the palliative treatment of lung cancer and has recruited over 1000 patients (Schiller *et al*, 2000, 2002). The primary endpoint was survival. There were no formal quality of life assessments or cost comparisons. Initially patients with performance status 0, 1, and 2 were included. However, the poor outcome and toxicity in the performance status 2 group resulted in this group being excluded from the trial (Johnson *et al*, 1999; Schiller *et al*, 2002). The four treatments compared were cisplatin/paclitaxel, cisplatin/gemcitabine, carboplatin/paclitaxel and cisplatin/docetaxel. Table 4

**Table 4:**
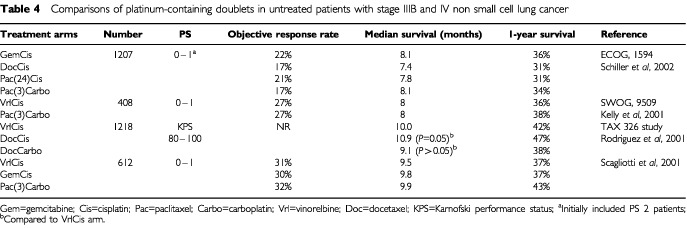
Comparisons of platinum-containing doublets in untreated patients with stage IIIB and IV non small cell lung cancer

shows the results. The response rates were of the order of 20%, the median survivals were all the same, and the percentage alive at 1 year was 31–36%. The time to progression was 1 month better for the cisplatin/gemcitabine arm, which reached statistical significance (*P*=0.001).

In the South West Oncology Group (SWOG), vinorelbine (25 mg m^−2^ week^−1^) and cisplatin (100 mg m^−2^ every 4 weeks) was considered the standard chemotherapy. Their 9509 study compared this arm with three-weekly paclitaxel (225 mg m^−2^) plus carboplatin (AUC 6), as used in the ECOG study. The objective response rate and median survival time were equivalent (27% and 8 months) and the 1-year survival similar (36% compared to 38%; *P* value, not significant) (Table 4). Quality of life was similar with approximately 60% of patients having improved or stable quality of life scores on both arms of the study. Toxicity appeared to be greater among patients receiving cisplatin/vinorelbine, with significantly higher rates of severe neutropenia, leucopenia, nausea and vomiting, whereas sensory neuropathy occurred significantly more frequently in the paclitaxel/carboplatin arm. This led to discontinuation of therapy due to toxicity in 28% of patients treated with cisplatin/vinorelbine compared with 15% of those receiving paclitaxel/carboplatin. However, the cost of treatment with paclitaxel/carboplatin is several fold higher (Kelly *et al*, 2001).

The cisplatin/vinorelbine regimen has also acted as the standard arm in two European studies, both enrolling patients of performance status 0 and 1, and both reported at ASCO 2001. The TAX 326 study was a large three arm trial in which the two other regimens examined were docetaxel (75 mg m^−2^) plus cisplatin (75 mg m^−2^) every 3 weeks and docetaxel (75 mg m^−2^) plus carboplatin (AUC 6) every 3 weeks. This trial demonstrates an improved quality of life with the lower dose of cisplatin but no statistically significant survival difference between arms after application of the Bonferoni correction for multiple statistical tests. The median survival was 10.9 months in the docetaxel/cisplatin arm compared with 10 months in the cisplatin/vinorelbine arm (*P*=0.05). The outcome in the docetaxel/carboplatin arm was not statistically different from that in the cisplatin/vinorelbine arm with a median survival of 9.1 months, and the rate of toxicity was lowest in this arm (Fossella, 2001; Rodriguez *et al*, 2001). The second study compared the same control arm of vinorelbine and cisplatin with two other doublets, gemcitabine (1250 mg m^−2^ days 1 and 8) plus cisplatin (75 mg m^−2^ day 2), and carboplatin (AUC 6, day 1) plus paclitaxel (225 mg m^−2^ day 1), each on a three-weekly cycle. The results are also shown in Table 4 and demonstrate no significant outcome differences between the three arms (Scagliotti *et al*, 2001).

### Doublets without platinum

The perceived toxicity of platinum drugs has also led researchers to investigate whether these can be omitted altogether. Two randomised trials have investigated the combination of paclitaxel and gemcitabine, and compared it with platinum-based doublets (Table 5

**Table 5:**
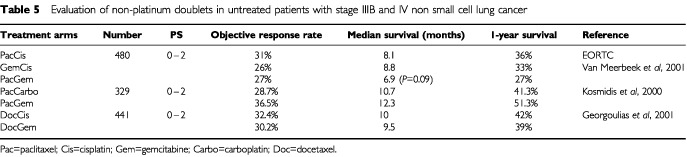
Evaluation of non-platinum doublets in untreated patients with stage IIIB and IV non small cell lung cancer

). An EORTC three-arm trial compared three-weekly paclitaxel (175 mg m^−2^ day 1) plus cisplatin (mg m^−2^ day 1) *vs* gemcitabine (1250 mg m^−2^ days 1 and 8) plus cisplatin (80 mg m^−2^ day 1) *vs* paclitaxel (175 mg m^−2^ day 1) plus gemcitabine (1250 mg m^−2^ days 1 and 8) (van Meerbeeck *et al*, 2001). The differences between these regimens did not reach statistical significance, but there was a trend towards a worse overall survival duration in the paclitaxel/gemcitabine arm. As expected, the rates of severe nausea and vomiting were lower in the paclitaxel/gemcitabine arm, but toxicity was otherwise similar. It should be noted that this trial was powered to detect a survival difference of 50% between regimens, and therefore, like many of the trials discussed, was underpowered to detect realistic small differences in efficacy. The second trial addressing this question was conducted in Greece, and compared three-weekly paclitaxel (200 mg m^−2^ day 1) plus carboplatin (AUC 6 day 1) with paclitaxel (200 mg m^−2^ day 1) plus gemcitabine (1000 mg m^−2^ days 1 and 8). This was also a relatively small trial, enrolling 329 patients, and no statistically significant outcome differences were observed. However, low rates of severe toxicity were seen in both arms of this study (Kosmidis *et al*, 2000). A third trial also conducted in Greece has compared the combination of docetaxel (100 mg m^−2^ day 8) plus gemcitabine (1100 mg m^−2^ days 1 and 8) with docetaxel (100 mg m^−2^ day 1) plus cisplatin (80 mg m^−2^ day 2). Four hundred and forty-one patients were randomised between the two arms, and all were supported with granulocyte colony stimulating factor during chemotherapy. Response rates were similar in the two groups (30.2% *vs* 32.4%) and no differences were observed in time to tumour progression or survival (Table 5). However, docetaxel plus gemcitabine had a more favourable toxicity profile with significantly lower rates of grade 3 or 4 neutropenia, nausea and vomiting and diarrhoea (Georgoulias *et al*, 2001).

### Two *vs* three drugs

A further question that has been addressed is whether adding a third drug to existing doublets might enhance their activity. Thus, triplets comprising cisplatin, gemcitabine and vinorelbine, and cisplatin, gemcitabine and paclitaxel have been compared with a cisplatin/gemcitabine doublet. Results are preliminary at present, but no clear advantage of the three-drug regimens has emerged to date, and toxicity is greater in comparison with this doublet (Comella *et al*, 2000; Alberola *et al*, 2001). Similar results have been produced in a comparison between the triplet vinorelbine, iphosphamide, cisplatin and the doublet vinorelbine/cisplatin, with no advantage for the triplet combination (Tan *et al*, 2001).

## ELDERLY PATIENTS WITH NON-SMALL CELL LUNG CANCER

Several trials have addressed the question of the optimal treatment for patients aged over 70 years. As discussed above in the section on vinorelbine, this drug was compared with best supportive care in this patient group and demonstrated palliative and survival benefits. Subsequently, single agent vinorelbine (30 mg m^−2^ days 1 and 8) was compared with a combination of vinorelbine (30 mg m^−2^ days 1 and 8) and gemcitabine (1000 mg m^−2^ days 1 and 8), with both regimens given on a three-weekly cycle. Planned enrolment was 240 patients, but the trial was stopped early following a planned interim analysis after the accrual of 152 patients, and analysis of response rates and survival for the first 120 patients entered in the trial. This demonstrated the superiority of the combination treatment, with a median overall survival of 29 weeks compared with 18 weeks in the single agent vinorelbine arm, and a relative risk of death after Cox multivariate analysis of 0.48 (95% CI 0.29–0.79; *P*<0.01). Toxicity was reported to show no significant differences between the two arms (Frasci *et al*, 2000). However, a second trial compared single agent vinorelbine with the combination of vinorelbine plus gemcitabine, and also incorporated a third arm comprising single agent gemcitabine. This trial included 698 patients and did not show any significant differences between the combination and either single agent in response rates, median overall survival or 1-year survival rates. Furthermore, this trial demonstrated higher rates of myelosuppression with the combination treatment (Gridelli *et al*, 2001).

## THE SECOND LINE TREATMENT OF NSCLC

Until recently, no agents have been available for use as a second line chemotherapy for lung cancer. Docetaxel has now been licensed for this purpose in Europe and in the USA. Two multicentre randomised trials have examined docetaxel in the second line treatment of lung cancer. The first trial included 373 patients and compared docetaxel to a control arm of vinorelbine or iphosphamide. Neither of these drugs would be considered standard second line treatment in this country. Docetaxel at a dose of 75 mg m^−2^ prolonged survival at 1 year (32% *vs* 19%, *P*=0.025), prolonged time to progression (*P*=0.046) and prolonged the progression free survival (*P*=0.005), but did not affect overall survival. Quality of life data has not been reported yet (Fossella *et al*, 2000).

The second trial including 104 patients, comparing docetaxel to supportive care alone, demonstrated a 2.4 month prolongation of survival with docetaxel plus supportive care compared to supportive care alone. Looking specifically at a docetaxel dose of 75 mg m^−2^ (which was better tolerated than 100 mg m^−2^) the median survival prolongation was 2.9 months (7.5 months *vs* 4.6 months; *P*=0.01) with an improved 1 year survival rate from 11% to 37% (*P*=0.003). Global health status assessed by the EORTC QLQ-C30 quality of life questionnaire favoured docetaxel. Outcomes were significantly better for pain and fatigue scales (*P*=0.006 and *P*=0.06 respectively), time to deterioration in performance status, and there was a decreased need for analgesia (*P*=0.01) and radiation therapy in the docetaxel arm. Toxicity with docetaxel was manageable and surprisingly not significantly different from patients receiving best supportive care alone (Shepherd *et al*, 2000). These improvements in outcome are comparable to those produced by irinotecan in the second line treatment of colon cancer (Cunningham *et al*, 1998), data that has been used to support the widespread adoption of this strategy in the UK (Table 6

**Table 6:**
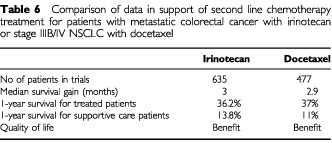
Comparison of data in support of second line chemotherapy treatment for patients with metastatic colorectal cancer with irinotecan or stage IIIB/IV NSCLC with docetaxel

).

## ECONOMIC ANALYSIS OF PALLIATIVECHEMOTHERAPY

The economic implications of palliative treatment for advanced NSCLC may be substantial as this is the commonest solid tumour in the UK and up to 40% of patients could become eligible for treatment in the next 3–5 years compared to an average of 8% currently. Most of the best health economic studies have been based in Canada. The POpulation HEalth Model (POHEM) is a model developed by the Health Analysis Modelling Group at Statistics Canada. This model looks at direct cost until death. Comparing cisplatin, doxorubicin and cyclophosphamide to best supportive care, the investigators showed a prolongation of life of the order of 8 weeks but with this there was a decrease in cost of the order of $949 (Canadian dollars) per patient treated with chemotherapy due to the decreased need for hospitalisation (Jaakkimainen *et al*, 1990). The cost of treating a patient with best supportive care in Canadian dollars is $140 895 and the cost with vinblastine and cisplatin, single agent vinorelbine, outpatient vinorelbine and cisplatin, and outpatient etoposide and cisplatin were all less than this. Thus these strategies are referred to as ‘dominant’ in that they prolong survival and were less costly per life year saved (LYS). Inpatient treatment was more costly than best supportive care (Evans and Le Chevalier, 1996). A similar US based study with a best supportive care arm showed again vinorelbine with cisplatin was cost effective compared to vindesine and cisplatin providing one LYS at a cost of $US15 500 (Smith *et al*, 1995). However, while this is valid for regimens that are low cost, or with new single agents when compared to no chemotherapy, it may not be the case when these new agents are combined with cisplatin as patients may still need hospitalisation, and overall the treatments may become very much more expensive. In the US-ECOG trial (Bonomi *et al*, 2000), which showed some superiority of either paclitaxel/cisplatin regimen over cisplatin and etoposide, the cost per life year saved (LYS) was an additional $30 619 for the use of the paclitaxel regimen (Earle and Evans, 1999). In the SWOG 9509 trial that demonstrated equal efficacy between the two treatment arms, an economic analysis at 24 months demonstrated that the majority of the cost difference was due to the additional cost of chemotherapy between the two regimens tested. This was $11 731 for paclitaxel and cisplatin and $3498 for vinorelbine and cisplatin for drug costs alone. All other supportive treatments e.g. blood products etc. were equal with the two treatments (Ramsey *et al*, 2000).

In Spain a combination of gemcitabine and cisplatin was no greater in total costs than MIC or cisplatin and etoposide (Cardenal *et al*, 1999; Sacristan *et al*, 2000). The cost of treating with chemotherapy compared to best supportive care translated from USA figures is about $15 500 per LYS which compares favourably with other health strategies e.g. dialysis for end stage renal failure at $53 000. It is also of the same order of magnitude as the use of taxanes for breast cancer, which when evaluated and approved by NICE was estimated to cost between £7000 and £24 000 per LYS. Health economic considerations change continually with changing practice in giving chemotherapy over time. For instance using rapid hydration to allow day case delivery of cisplatin reduces bed occupancy, or conversely the use of growth factors leads to higher costs.

There is only one UK study which details bed occupancy which is the major non drug expense and this is the Manchester based study comparing best supportive care to gemcitabine. Patients receiving best supportive care required on average 2012 days in hospital and 247 in hospice compared with 1746 days in hospital and 183 days in hospice for gemcitabine treated patients. In addition the gemcitabine treated patients required less radiotherapy (Anderson *et al*, 2000). This is of particular importance in the UK where delays in radiotherapy delivery are commonplace due to limited machine time. Second line treatment will be a new added treatment cost with continuing drug development, particularly if the promising new biological agents are brought successfully into clinical practice.

## CONCLUSIONS

The National Institute for Clinical Excellence (NICE) has reviewed the use of chemotherapy for the palliative treatment of non small cell lung cancer in the year 2001. The volume of data on the use of chemotherapy, both new and old, is impressive. Unlike the treating doctors in the past, the committee felt that a prolongation of survival by 6–8 weeks in a life that is predicted to be 6 months is important. The beneficial effects on quality of life and symptom control further increase the value of this treatment. Their evaluation of the new chemotherapy agents docetaxel, paclitaxel, gemcitabine and vinorelbine for this disease was therefore in function of this and their recommendations were that these drugs should be considered treatment options in accordance with their licences. Thus paclitaxel, vinorelbine and gemcitabine are all considered reasonable first-line treatment options in combination with cisplatin, and single-agent docetaxel is considered a suitable second-line therapy. They concluded that the current evidence is insufficient to allow recommendation of a specific first-line regimen as superior to the alternatives, but that patient and carer preferences as well as economic considerations should play a part in determining the appropriate treatment for an individual.

There remains considerable controversy as to whether the new drug combinations provide a significant advance over established UK regimens such as MVP and MIC, largely due to the lack of randomised clinical trials of sufficient power comparing these treatments. The results in terms of the percentage of patients alive at 1 year using MVP and MIC in UK-based phase III studies are included in Table 3. The disparity between these figures and those produced in recent trials of the new generation doublets could be due to differences in the patient populations between the UK trials and those conducted in North America and the rest of Europe. Alternatively, MIC and MVP may be truly less active regimens. For a two-arm trial to show a 5–8% difference in 1-year survival rate approximately 1000 patients would be required, and such trials have not been performed to date. Currently the market for these new agents is small in the UK compared to the market worldwide. It is therefore unlikely that the major pharmaceutical companies will fund these initiatives unless there is a suggestion that if the new treatments prove better they will become widely used and available. However, if we in the UK wish to move forward we cannot stall any more, and now have the mandate to treat. As more trials are reported and initiated we must have more money in the system so that the UK can be a participant or leader – this will not happen if we do not have regimens other than MIC and MVP on our menu of treatment options.
